# Commentary: A Pilot Digital Intervention Targeting Loneliness in Youth Mental Health

**DOI:** 10.3389/fpsyt.2019.00959

**Published:** 2020-01-24

**Authors:** Kelly-Ann Allen

**Affiliations:** Educational Psychology and Inclusion, Faculty of Education, Monash University, Clayton, VIC, Australia

**Keywords:** smartphone, mental health, psychosis, belonging, loneliness

## Introduction

Positive relationships are fundamental to our physical and psychological health and wellbeing, yet loneliness is now estimated to affect one in four members of the general population to the detriment of their physical and psychological health (1–4). To combat rising rates of loneliness, a number of interventions are available that focus on increasing social connections. However, as Lim et al. (5) suggests, increasing the quantity of relationships is not, by itself, enough. In order to improve feelings of loneliness, it is also necessary to address the *quality* of relationships. This is the central aim of the +Connect smartphone app. The pilot of +Connect offers exciting preliminary insights into the delivery of an intervention for loneliness in young adults (5). However, design choices in a number of areas—including the digital platform used in the intervention and a focus on current relationships—set boundaries on the utility of the current iteration of the app. I argue that these additional considerations should be reflected upon in the design of future iterations of this app and that Lim and colleagues (5) can fruitfully draw from empirical work in related areas (e.g., belonging, social skills, cognitive-based interventions, and motivation) to create more diverse and tailored offerings.

### An Intervention for Loneliness

+Connect (5) was created as a 6-week digital intervention program with the goal of a) targeting loneliness by improving relationship quality among young adults with Social Anxiety Disorder (SAD) and b) extending past research (6, 7). The pilot intervention employs a positive psychology approach to help young adults identify their strengths, increase their positive affect, and focus on building intimacy within existing relationships. The intervention yielded positive results, proving to be beneficial. An impressive 75%–100% of participants with SAD reported +Connect to be helpful. However, the researchers also noted a high attrition rate for participants with SAD, which was almost double the rate of those without the disorder (5).

An important strength of the app is its use of shared experience videos. The designers of the app have identified and made valuable use of an opportunity to normalize the experience of loneliness as a commonly occurring human emotion. This insight offers an alternative perspective to recent scholarly work that has sought to pathologize loneliness (8).

Another strength is that the app offers an alternative to medical approaches that posit that loneliness is a condition that requires pharmaceutical treatment (9). +Connect provides a non-medical, psychologically based intervention that offers users skills that also benefit the general population, irrespective of their mental health status or even whether they are lonely or not (5).

### Loneliness and Belonging

Lim and colleagues (5) emphasize that loneliness does not always stem from being alone or physically isolated and they rightly point out that a successful intervention does not simply involve recruiting people to join groups. Instead, the authors suggest that increasing the satisfaction with, and perceived meaning of social connections in addition to focusing on the quality of interactions, rather than the quantity, offers a more helpful approach to addressing perceived loneliness. However, while the aim to improve the quality of current relationships offers considerable value, the focus on *current* relationships is limiting in that it offers little help for those who are unable to make substantial social connections in the first place. Quantity is not enough by itself, nor is quality. Both are needed together. Other research areas in the empirical literature point toward ways in which this focus could be broadened to improve successful outcomes from the app [e.g., (10–18)]. The +Connect app may also be beneficial for other at-risk youth, such as young people who have a parent with mental illness who indicate that they want more support through digital technology (19).

Loneliness and belonging, for example, are considered by many scholars to be distinct but related subjective states that play a role in normal human emotion (20, 21). According to this kind of approach, belonging is a psychological need for interpersonal bonds [e.g., (11)], while loneliness results from the perceived discrepancy between an individual’s actual and desired social relationships (22). When needs for belonging are not met, this leads to perceived loneliness (21). There is evidence that, because loneliness and belonging share similar characteristics and are highly correlated, an increase in feelings of belonging will improve feelings of loneliness (20). Conversely, supporting belonging is associated with decreased loneliness (21). Interventions targeting belonging may, thus, offer rich and new insights and perspectives into ways in which loneliness can be addressed (23).

A more balanced and well-rounded intervention might also consider the inclusion of social skills training [to address those users who may be lonely as a result of social skills deficits or limited interpersonal skills; see (10, 13)], cognitive-based interventions [to address those users whose perceptions of their experience of loneliness may be irrational or due to fundamental attribution errors; (17, 18)], and motivation [assuming that some users simply lack the motivation to create more meaningful connections; (11, 15)]. We are seeing increasing evidence for the success of these types of interventions delivered through online technologies for various outcomes [e.g., Growth mindset and social belonging, (12); treating depression, (14)]. However, more research is needed before this approach could be validated as a useful inclusion in the +Connect app. The inclusion of more diverse interventions offers routes by which to reach more people, offer greater variety for users, and allow for the app to engage with a broader audience. It is well established in the literature that positive psychology interventions, such as those included in the +Connect app, are more effective when factors such as personal fit, variety, and dosage are considered in respect to the individual personal features of the user (16). Diversity in intervention delivery may also offer additional ways of developing the cross-cultural applicability of the insights underlying the app.

### Current Social Media Apps

In evaluating the +Connect app, it is necessary to ask whether and to what extent it is capable of doing more than non-specialized, commonly used social media apps (such as Instagram, Twitter, Facebook, or TikTok) that have been found in some research to create opportunities to connect with others and reduce loneliness (24–27). While social media may create digital communities (28), there are good reasons to think that the connection formed does not create the same level of satisfaction that comes from the face-to-face relationships that the +Connect app aims to enhance through specifically improving the perceived quality of relationships. Some recent research also suggests that social media use can *add* to feelings of loneliness and isolation [e.g., (29)] although other studies suggest that these types of finding are highly dependent on the user and the context, including the frequency of use (16, 30). +Connect differs from common social media apps in that it aims to *build* skills with the goal of increasing social support systems. It also sidesteps various problems for mental health and wellbeing that research has associated with the use of other social media apps, such as criticisms related to cyberbullying, addiction, and unrealistic social comparisons (31–34). Pittman and Reich (26) have demonstrated that social media platforms based on video and picture content can actually enhance a sense of connection with others, while other research has shown the benefits of gamification for wellbeing interventions (35). The core use of video technology and an interactive platform in +Connect is, thus, likely to be another strength of the app.

### Limitations

A central limitation of the +Connect app is that it may not reach all people who could potentially benefit from it, such as those without phones and those who are socially isolated. While the vast majority of young people in developed countries own phones, many still do not. This fact alone excludes a significant number of individuals from receiving help and support for feelings of loneliness and isolation. The focus of +Connect on increasing the quality of *existing* relationships also sidelines those who do not currently have such relationships or who are looking to explore new relationships.

Another possible concern with the primary platform chosen to deliver the +Connect content is that the very technology it uses has been identified by some scholars as problematic for loneliness (27, 28, 36–38). While +Connect aims to address loneliness in young adults, it may also unwittingly promote increased digital screen time and reduced time spent on face-to-face connections.

### Future Research and Considerations

It may be possible to explore other ways of connecting in order to expand upon +Connect’s goal of reaching more people, such as through school programs, university electives, and community-based programs. The core content of the +Connect intervention is delivered through videos. It should, thus, be feasible to consider that this content might usefully be delivered through different platforms in addition to delivery through the app itself, an exclusive focus on which may prove to be unnecessarily restrictive.

Future research should address issues with the current intervention and methodology in order to provide more in-app tailored support to address attrition rates of users with mental health difficulties, an investigation into potential use for other age groups who have been identified as most at-risk for loneliness [i.e. adolescence and older adults (2)], cross-cultural applicability, suitable alternatives for people without phones, opportunities for those looking for new relationships, and larger samples for generalizability. An important next step will be a pilot randomized controlled trial.

## Conclusion

+Connect was developed with the goal of addressing loneliness in young adults and was found to be beneficial for the participants in the study, especially with regards to their overall quality of life. The findings suggest that those with SAD may benefit from such interventions but require tailored support to reduce dropout rates. Lim et al. (5) demonstrate sufficient evidence for the value of further work to examine the effectiveness of +Connect within a pilot randomized controlled trial. This commentary has argued that the app itself could be strengthened by drawing from other fields of research and meeting the needs of a broader audience, especially those without access to digital platforms and those who are physically alone, socially isolated, or ostracized. Interventions such as +Connect show great preliminary promise for targeting loneliness and should be extended urgently to explore utility with other age groups, cultural groups, and with alternative modes of delivery. +Connect plays a role in signaling the urgent need to evaluate systemic factors that may enhance or hinder loneliness in society (e.g., legislation, town planning, architecture, medical approaches, and general public awareness of the importance of belonging to groups and the detrimental effects of chronic loneliness). Lim and colleagues (5) have been proactive in raising awareness of the need for more funding and research for apps like +Connect that are preventative and proactive. Approaches that seek to address loneliness through a systemic lens are also concurrently needed in addition to interventions such as +Connect in order to offer a broad-based approach to the problem of loneliness moving into the future.

## Author Contributions

The author confirms being the sole contributor of this work and has approved it for publication.

## Conflict of Interest

The author declares that the research was conducted in the absence of any commercial or financial relationships that could be construed as a potential conflict of interest.
